# Medical and physician assistant students’ views on integrating comics into medical education

**DOI:** 10.15694/mep.2017.000196

**Published:** 2017-11-03

**Authors:** Amani Elghafri, Renee R. Stewart, Ramya A. Sampath, Jennifer C. Kesselheim, Michael J. Green

**Affiliations:** 1 Internal Medicine Residency Program; 2 Department of Humanities; 3 Division of General Internal Medicine and Primary Care; 4 Master of Medical Sciences in Medical Education Program; 5 Departments of Humanities and Medicine

**Keywords:** Comics and Medicine, Medical Education, Educational Comics, Medical Ethics, End of Life Care

## Abstract

This article was migrated. The article was marked as recommended.

***Purpose***: This study explored comics as a tool for teaching medical and physician assistant (PA) students about end-of-life decisions and advance care planning.

***Methods:*** Using a mixed method convergent design, a survey (consisting of a five-point Likert scale and open-ended questions) was administered to second-year medical and first-year PA students enrolled in an Ethics and Professionalism class at a US medical school. The survey assessed students’ perspectives on the addition of a comic “Betty P.” to assigned readings and about the use of comics in the classroom. Quantitative results were compared by demographics, and open-ended responses were analyzed qualitatively for emergent themes. Quantitative and qualitative findings were compared for correspondence.

***Results:*** Of the 145 students who completed the survey (83%), 141 students (81%) had read the comic. The vast majority (89%) felt that “Betty P.” helped them understand end of life care for patients, and 84% felt that the comic did not distract them from the seriousness of the subject. Qualitative analysis revealed 2 major themes: 1) comics were educational, and 2) comics engaged learners emotionally. We observed convergence between quantitative and qualitative results.

***Conclusion:*** Integrating comics as a supplemental teaching tool is an innovative way to engage medical students.

## Introduction

The use of comics has been shown to improve students’ learning experiences in a broad range of disciplines ( Gerde and Foster, 2008; Muzumdar and Nania, 2015) Comics are increasingly being incorporated into medical school curricula in light of promising research demonstrating their ability to convey experiences and information not found in other types of educational materials ( Green, 2013; Joshi et al., 2015). One recent study suggests that comics can help portray illness and the realities of medical practice from myriad viewpoints not often found in medical textbooks and can help medical students gain a more holistic understanding of the practice of medicine than through texts alone ( Green, 2013). As Paul Gravett, a journalist and comic book author, noted in his keynote address at the 2011 Comics and Medicine Conference, “the reason I am drawn to comics is empathy, being able to understand other people’s lives and experiences and hopefully finding a connection in yourself. Even if it is something you’ve never experienced, you can open up your mind and heart to what other people are going through” ( Gravett, 2011). Comics’ potential to facilitate empathy and understanding may be particularly relevant for teaching about ethics and end-of-life (EOL) care, as recent studies have shown that medical students and interns are not adequately prepared to address EOL issues with patients ( Buss et al., 1998; Ury et al., 2003; Siu et al., 2010).

Although there is some evidence that comics can be effective teaching tools in medical education, they have not been evaluated in the context of teaching about medical ethics and EOL care ( Muzumdar and Nania, 2015; Hosler and Boomer, 2011; Kraft et al., 2017). The purpose of this study was to explore the impact on students’ experiences of integrating comics into a medical school ethics course during a session devoted to EOL care. We hypothesized that including the short, published graphic narrative “Betty P” as a supplement to text-only readings would better engage students in the themes of the course and help them gain a deeper understanding of both physicians’ and patients’ experiences ( Green and Rieck, 2015).

## Methods

### Study design

**Figure 1.**
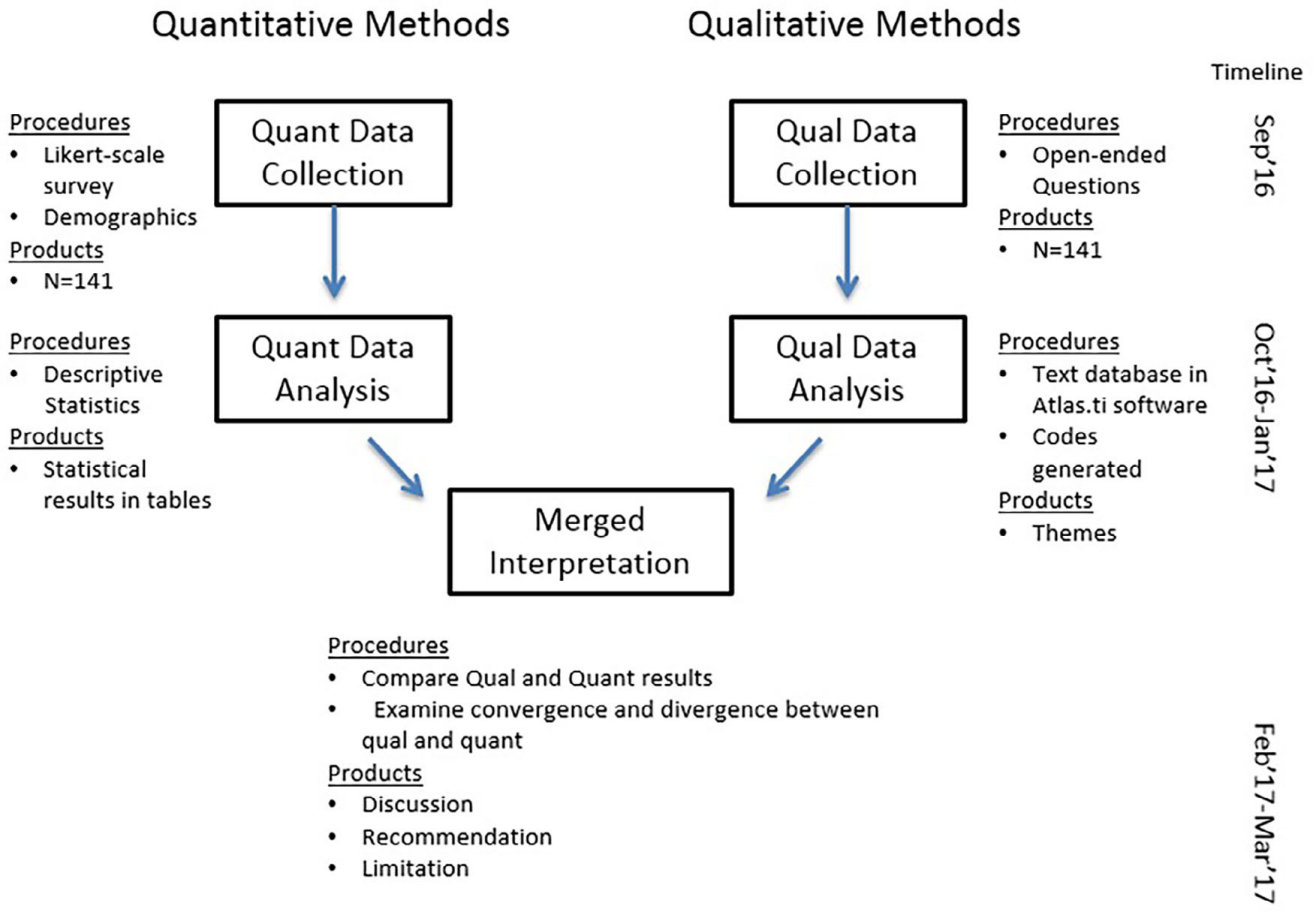
A convergent parallel mixed methods design including overview of timeline, methods, and results from a study of medical and physician assistant students’ attitudes toward the use of comics in an Ethics and Professionalism Course at Penn State College of Medicine, 2016

This study used a mixed methods approach (see Fig. 1) in which we collected quantitative and qualitative data simultaneously during a one-week phase. ( Schifferdecker and Reed, 2009; Creswell, 2015). Results were integrated at the final analysis, in which we assessed convergence and divergence between the qualitative and quantitative results ( Yin, 2006).

### Participants and Setting

The study population consisted of all second-year medical students and first-year physician assistant (PA) students enrolled in a mandatory Ethics and Professionalism course at Penn State College of Medicine in Hershey, Pennsylvania in 2016. Comprising 12 sessions, the course met weekly for a one-hour plenary session after which students separated into small groups of nine to ten students for an hour of discussion. The course covered a variety of medical ethics topics, and students were asked to complete preparatory readings in advance of each class.

### Intervention

“Betty P.” is a five-page comic written by Dr. Michael J. Green (author MG), a physician and bioethicist, and illustrated by Ray Rieck ( Green, 2015). Peer-reviewed and published in The Annals of Internal Medicine, “Betty P” raises questions concerning medical ethics and EOL care. The narrative centers around a dramatic situation faced by the author during his clinical training when he participated in a futile cardiopulmonary resuscitation attempt on a terminally ill patient who lacked an advance directive.

“Betty P.” was assigned to students as a required reading along with two other peer-reviewed articles and a short video on EOL care ( Balaban, 2000; Lantos, 2004; FORA.tv, 2017). As was usual for the course, students convened for a one-hour lecture and then divided into small groups to discuss the lecture and readings, including the comic.

### Survey development

The authors (AE, JK, MG) developed and pilot-tested the survey questionnaire for face validity and clarity with a convenience sample of three third-year medical students and three course facilitators. Based on this feedback, the researchers refined the questions through an iterative process.

The survey consisted of four sections: 1) two pre-qualifier dichotomous questions; 2) eleven attitudinal questions on a five-point Likert scale (where 1=strongly disagree and 5=strongly agree); 3) three demographic questions; and 4) four open-ended questions. The purpose of the first pre-qualifier dichotomous question was to exclude those who did not read the comic from completing the survey. The full text of the questions is included in Table 1.

**Table 1.** Survey questions regarding perceived educational value of “Betty P.”

Survey Questions	Answer Type
Did you read the comic “Betty P.”?	(Yes/No)
Did you read the comic “Betty P.” carefully or just skim it?	
What (if anything) did you learn or appreciate from the comic “Betty P.” that you might not have learned/appreciated from a typical text-only reading?	Open-ended
In what way did the comic format enhance or detract from your appreciation of the subject matter?	
Would you like to have more comic readings in other subjects? Why/why not?	
Any other comments?	
Compared to the text readings, the comic: a. Gave me a deeper appreciation of the importance of effective communication with patients at the end of their life b. Was more effective at helping me understand patients’ right to decide their end-of-life care plan c. Helped me better understand the consequences of not completing an advance directive with terminally ill patients d. Helped me participate more actively during small group discussion e. Distracted me from focusing on this serious subject matter f. Helped me to understand the importance of discussing end-of-life care with my patients	5-point Likert Scale
How do you think including more comics as part of your assigned readings would impact your: a. Engagement with subject matter b. Motivation to learn c. Compassion for the patient d. Empathy towards others e. Confidence in approaching ethical issues or decisions	5-point Likert Scale

### Data collection

Students were informed of the study during the plenary session devoted to the topic of advance directives and EOL care (September 13, 2016), and invited to participate via a post-class online survey. After the lecture and small group discussion sessions ended, students were sent an email, which included a description of the study and link to the online survey. Participation was voluntary and unrelated to course grades, and completion of the survey implied consent to participate. As an incentive to participate, students were entered into a raffle to win a Kindle e-reader upon completion of the survey. Responses were anonymous and collected via Qualtrics during a single week in September 2016. The project was reviewed and approved by IRBs at Harvard Medical School and Penn State Milton S. Hershey Medical Center.

### Quantitative analysis

We analyzed students’ responses to the 11 Likert-scale questions, calculating means and standard deviations of responses on the five-point scale. We also calculated percentages of students who agreed or disagreed by dichotomizing responses as “agreement” (4 or 5 on the scale) or “disagreement” (1 or 2 on the scale).

### Qualitative analysis

We qualitatively analyzed students’ views about the use of comics as reported in the survey’s open-ended questions. Using an inductive approach, two researchers (AE, RRS) generated a codebook in ATLAS.ti 8 software. They independently open-coded the first question’s responses and then compared their work, discussing how to proceed with the rest of coding. Any identified discrepancies were resolved via discussion until full inter-coder agreement was reached. The 37 codes generated from the data were compared and collapsed to 20 codes and then further refined into 5 codes. Two researchers (AE, RAS) further distilled these codes into two major themes.

## Results

### Participants

A total of 145 of 174 eligible students completed the survey, yielding a response rate of 83%. Of those who completed the survey, 141 (97%) students indicated that they read the comic before lecture and were therefore included in further analysis. Of these 141 students, 123 (87%) reported that they had read the comic carefully.

The final sample consisted of 114 second-year medical students and 27 first-year physician assistant students. As shown in Table 2, 85 (60%) of the participants were females and 99 (70%) were white. Participants’ ages ranged from 21 to 34 with a mean age of 25 years (SD 2.3).

**Table 2.** Demographic characteristics of students who read “Betty P.” in an Ethics and Professionalism course at Penn State College of Medicine, 2016.

Characteristics	n = 141
Age, y	
**Mean (SD)**	25 (2.3)
**Range**	21 - 34
Gender, no. (%)	
**Male**	56 (40)
**Female**	85 (60)
Race, no. (%)	
**White**	99 (70)
**Not White**	36 (26)
**Not specified/others**	6 (4)

### Quantitative outcomes

A vast majority of participants (89%) felt that “Betty P.” helped them better understand EOL care situations and decisions with patients, and 84% felt that the comic did not distract them from focusing on the seriousness of the subject at hand. Further, 84% indicated that adding comics to future pre-course readings would increase their engagement with subjects they were learning. About half of students (48%) believed that reading the comic increased their participation in class discussion. Complete quantitative results are presented in Table 3.

**Table 3.** Students’ responses (n=141) to a Likert-scale survey regarding the perceived impact of the comic “Betty P.” on their understanding of topics presented in an Ethics and Professionalism course at Penn State College of Medicine, 2016.

Questions	Mean (SD)	% Agreement, n
***1. Compared to the other assigned text readings, the comic:***		
**a. Gave me a deeper appreciation of the importance of effective communication with patients at the end of their life**	3.9 (0.8)	75%, n=109
**b. Was more effective at helping me understand patients’ right to decide their end-of-life care plan**	3.6 (0.9)	56%, n=79
**c. Helped me better understand the consequences of not completing an advance directive with terminally ill patients**	4.3 (0.7)	89%, n=126
**d. Helped me participate more actively during small group discussion**	3.5 (0.9)	48%, n=68
**e. Distracted me from focusing on this serious subject matter**	1.9 (0.9)	16%, n=22
***2. How do you think including more comics as part of your assigned readings would impact your:***		
**a. Engagement with subject matter**	4.0 (0.7)	84%, n=119
**b. Motivation to learn**	3.7 (0.8)	62%, n=87
**c. Compassion for the patient**	3.9 (0.7)	74%, n=104
**d. Empathy towards others**	3.7 (0.7)	56%, n=79
**e. Confidence in approaching ethical issues or decisions**	3.4 (0.6)	39%, n=55

### Qualitative outcomes

Two main themes emerged from the qualitative analysis: 1) comics were educational, and 2) comics engaged learners emotionally.

### Theme 1: Comics as educational media

i. The comic was an effective tool for conveying information

Numerous participants suggested that the comic was effective at communicating complex ideas and details around EOL care.

*“I particularly appreciated the weight of the moment where [the doctor] is doing CPR and hearing and feeling the ribs crack, because it’s paired with a facial expression that heightens my experience of what he is going through. I think that really drove home the message that unnecessary lifesaving procedures can be harmful both to the people receiving them and the people giving them”* 25, male, physician assistant student (PA)

Students reported that the comic helped them more quickly understand the information presented than they could have through text alone, which they attributed to the visual quality and conciseness afforded by the format.

*“It was short, sweet, and to the point. The same information written in prose and without pictures would have taken longer to read and not stuck as well in my memory.”* 23, female, medical student (MS)

Several participants discussed the suitability of comics for catering to a visual learning style. Being able to visualize the scenario helped students understand what was at stake for the doctor in the story.

*“I am a visual learner, so the comic just made the situation a lot more ‘real’ to me. It was more impactful to see the anguished face of the student as he performed CPR that he thought was inappropriate.”* 24, female, PA

ii. The comic facilitated experiential learning

By depicting the impact of CPR on this terminally ill patient and her doctor, the comic created a narrative realism that would not have been achievable through text alone.

*“I really like the comic format because it made the reading seem like less of an assignment and more of a real-life situation. It allowed me to appreciate the situation more because I saw faces along with the text.”* 26, female, PA

Further, many students reported that given their limited clinical exposure, they were better able to visualize the situation and imagine themselves in it.

*“It made it easier to visualize the patient and all the trauma inflicted on her by the CPR. A lot of medical students haven’t had experiences dealing with patients yet. Trying to visualize a scenario on our own sometimes is hard if we have nothing to compare it to.”* 23, female, MS

iii. The comic provided a valuable complement to text

Several students reported feeling that the comic helped reinforce concepts presented in their text readings, sometimes surpassing text in its capability to depict emotions and important takeaways.

*“I thought I understood the importance of having an advanced directive/encouraging others to have one well before it would be used, but this comic really brought that importance to the forefront and got its point across more so than any article I have read on the subject.”* 24, female, PA

Some students suggested that using multiple learning media can create a more robust learning experience.

*“I think approaching a subject with multiple methods (normal reading, video, comic) is superior than a single method (just reading). It gives a more complete understanding of a subject.”* 23, male, MS

iv. Comics have certain limitations

A few participants suggested that comics may be better suited for teaching some subjects over others. They conjectured that topics within humanities might be better suited for comics than other, more scientific fields of medicine.

*“I think it may depend on the subject matter. Having comics in more clinical or scientific subjects may detract from the overall learning of the topic.”* 23, male, MS

A small number of students expressed skepticism about the educational value of comics as medical education tools more broadly.

*“I feel it can take away some of the depth that can be put in if it were just straight text.”* 27, male, MS

### Theme 2: Visual learning through emotional engagement

I. The comic elicited an emotional response which aids learning

Many students highlighted the significant role the comic had in depicting emotions and eliciting an emotional response in the students. This emotional response, they suggested, had a role in their learning about the importance of advance care planning.

“ *The ability to view the emotions of the characters through the illustrations created a strong emotional response that I feel will stick with me as I move into my career. If I ever find myself in a situation similar to the one experienced by the resident in the comic, then I might recall this story and be able to take some sort of action before the outcome becomes a negative one.*” 24, male, MS

II. The comic fostered empathy for the characters

Many students felt that the comic’s depiction of characters’ emotions throughout the narrative enabled them to better connect with the characters. They were able to envision themselves as the doctor in the narrative and empathized with him for the difficult choices he had to make in an ethically complex situation.

*“The physician’s emotions as he performed chest compressions on his fragile, elderly patient were much more ‘moving’ and evident, and I believe the artwork additionally made the story more engaging. The characters’ struggles seemed more relatable, and I think the artists’ skill in depicting his/her characters’ emotions through their facial expressions made it easier to empathize with the characters involved.”* 25, female, MS

### Correspondence between quantitative and qualitative outcomes

We observed several areas of convergence between the quantitative and qualitative data. The vast majority of students responded with a 4 or 5 (agree or strongly agree) on survey questions that pertained to the comic’s ability to stimulate engagement and understanding of the ethics and EOL concepts presented in class. Most students also expressed enthusiasm for the educational value of “Betty P.” and comics more generally in the open-ended questions.

Less than half of students (39%, n=55) felt that the comics could increase their confidence in ethical decision-making (response of 4 or 5 on question 8e). Similarly, in the open-ended questions, although students frequently discussed the comic’s impact on their engagement, understanding, and emotional experience, they rarely commented on how this would translate to confidence in a real life situation. We did not observe divergence between the quantitative and qualitative data.

## Discussion

Using “Betty P.” alongside text-only readings in their class, students reported better understanding the importance of discussing EOL care with patients, the role of advance directives, and the value of effective communication. Rather than viewing the comic’s format as a distraction from the seriousness of the topic at hand, most students found it to be an accessible way to address a complex ethical topic. Students felt that “Betty P.” conveyed emotional and situational nuance that would be difficult to do with text alone.

Medical educators appropriately expect students to acquire information from reliable and scholarly sources, and our research suggests that comics could be valuable teaching tools in medical education for explaining complex subjects efficiently and quickly. Further, fields such as narrative medicine and anthropology have long recognized the pedagogical value of narrative for illustrating important themes or aspects of individuals’ experiences (Charon, YEAR; McNicol, 2014). By visually depicting narratives, comics may help students bridge the gap between classroom instruction and experiential learning, helping to forecast the experiences of clinicians and patients from within a classroom setting.

Some students speculated that humanities concepts were better suited for graphic representation than concepts in basic science. Research shows that for comics to effectively supplement textual media they must reflect the complexity of the ideas and language presented in the text ( Williams, 2012; Khoii and Forouzesh, 2010). Curriculum developers are experimenting with using comics to convey basic science concepts as well. For example, JP Medical’s “Eureka” series combines detailed molecular, physiological, and anatomical illustrations with holistic depictions of patients, illustrating aspects of the patient’s experience, presentation, diagnosis, and management of medical conditions (the3ateam, 2015). To date, there have been no systematic evaluations comparing the efficacy of such texts with traditional learning materials. Such comparative work would be valuable for assessing the role of basic science comics in the medical curriculum.

### Limitations

This study was not randomized, and there was no control group for comparison. Only one sample population over a short period of time was observed and this could limit generalizability of our findings. However, this study could easily be replicated at other medical schools using randomization with “Betty P.” or some other comic to supplement traditional educational methods.

Second, without longitudinal data, we cannot determine the long-term impact of this comic. Future research could follow a cohort of students into their first years of clinical practice to determine if the particulars of the comic and its lessons stayed with them and shaped their clinical decision-making.

Third, the study was not designed to assess actual knowledge of the concepts presented, rather it focused on students’ subjective perspectives and experiences reading the comic. To know whether students actually gained better understanding of the concepts presented in the course would require pre-and post-tests assessing understanding.

## Conclusions

The addition of the comic “Betty P.” into the medical school curriculum helped students more deeply engage with the moral and clinical complexities of end-of-life care. Students strongly supported the additional integration of comics into the medical school curriculum.

This study contributes to a growing body of research examining the role of comics in medical education to enhance learning outcomes and to help students better visualize clinical situations. By directing resources to developing comics-based case studies for other topics in medicine and researching their effectiveness, medical institutions will be able to create and evaluate the use of new media to engage learners.

## Take Home Messages

• “Betty P.,” the comic explored by this study, helped students intellectually and emotionally engage with concepts in bioethics and EOL care.
• Students are eager for creative, new tools to be introduced into medical curricula to enliven abstract concepts and convey situational nuance.

## Notes On Contributors

**A. Elghafri, MD, MMSc** is a graduate of the Medical Sciences in Medical Education Masters Program at Harvard Medical School, and she is a resident in Internal Medicine at Beaumont Hospital, Dearborn, Michigan. She is passionate about innovation in medical education, particularly the use of graphic novels.

**R.R. Stewart, MS** is a research coordinator in the Department of Humanities at Penn State College of Medicine in Hershey, PA. She collected and analysed both quantitative and qualitative data for a study of surrogate decision-making during advanced illness. She received her Masters of Sciences in Public Health Sciences from Penn State Hershey.

**R.A. Sampath, BA** is a Fulbright-Nehru Fellow at the Trivandrum Institute of Palliative Sciences in India. She has conducted research at Brigham and Women’s Hospital on bioethics and medical decision-making during advanced illness and has supported teaching and curricular change at universities in the US and Egypt. She studied anthropology at the University of Chicago.

**J.C. Kesselheim, MD, MEd** is the founding director of Harvard Medical School’s Master of Medical Sciences in Medical Education Program. She is a pediatric hematologist-oncologist and a Harvard Medical School Assistant Professor of Pediatrics at the Dana-Farber Cancer Institute and Boston Children’s Hospital.

**M.J. Green, MD, MS** is a physician and bioethicist at Penn State University’s Milton S. Hershey Medical Center. He is Interim Chair of the Department of Humanities, Chair of the Hospital Ethics Committee, and Director of the Program in Bioethics. He is also an innovator in the use of comics in medical education.
