# Supplementary material for: Medical and physician assistant students’ views on integrating comics into medical education
Source: MedEdPublish (2016). 2017 Nov 3;6:196. [Version 1] doi: 10.15694/mep.2017.000196 (PMC10885315; doi:10.15694/mep.2017.000196)
Supplement: Peer review history

# Reviewer report: Reviewer response for version 1

**DOI:** 10.21956/mep.19382.r28400

**Published:** 2019-04-18

**Authors:** Ken Masters

**Affiliations:** 1 Sultan Qaboos University

This review has been migrated. The reviewer awarded 3 stars out of 5 An interesting paper on a somewhat novel topic of using comics in the training of medical and physician assistant students, specifically in medical ethics and EOL care. The authors have provided a clear background of the literature and the setting. They have correctly noted that they have not followed up on the students’ learning, but the paper is focused on student perceptions, and so measures that well enough – using both quantitative and qualitative data helps to shed light on the students’ rationale behind the answers.One issue• The authors gathered demographic data, but then, apart from simply listing the data in Table 2, never refer to them again. It is unclear as to why these data were gathered if never used (e.g. checking to see if there were differences in perceptions between gender or race). Given that they already had these data, it would have been more valuable if the authors had conducted a few simple tests to see if there were any differences among sub-groups.Two small points• In the Abstract: “Of the 145 students who completed the survey (83%), 141 students (81%) had read the comic”. The figures of 141/145 don’t match the percentage of 81% (and conflict with the figures presented in the body of the paper).• There is a small error in the citing of “experiences (Charon, YEAR; McNicol, 2014).”An interesting read, on a topic that could benefit from further research.

Reviewer Expertise:

NA

No decision status is available

# Reviewer report: Reviewer response for version 1

**DOI:** 10.21956/mep.19382.r28399

**Published:** 2017-11-08

**Authors:** Gerard Flaherty

**Affiliations:** 1 National University of Ireland Galway

This review has been migrated. The reviewer awarded 4 stars out of 5 I enjoyed reading this novel manuscript. The authors provide a balanced rationale for the use of comics as a supplementary educational tool in health professional ethics education. Their mixed methods study is well designed and the limitations relating to non-randomisation and lack of a control group are acknowledged. The survey questionnaire, though piloted, is not validated and reference should be made to this. Is it possible, for example, to yield values for a reliability coefficient from the quantitative data obtained? The description of the qualitative analysis of the free text responses is rigorous and the coding procedures robust. It is refreshing to see the apparent value of comics in promoting student engagement. It is not possible in the current study to determine the durability of the comics' impact on the learner, nor whether students who identify themselves as visual learners benefited to a greater extent than more verbal learners. This may be a useful follow on research project. I did wonder how much this learning approach might depend on the skill of the illustrator but there may be useful opportunities for collaboration between different university departments in producing authentic learning material in this respect. Comics, if well illustrated and adapted into a narrative, could be regarded as a form of virtual reality, but the discrete nature of comic 'frames' might help to underscore certain teaching points, such as the ethical dilemmas faced by the person providing CPR in the comic studied. In this regard, comics may have advantages over continuous videos, where facial expressions are fleeting. Certain students, such as those on the autism spectrum, might find the opportunity to carefully study facial expressions in a comic format particularly helpful. Overall, this was a very creative and well executed study which provided ample food for thought.

Reviewer Expertise:

NA

No decision status is available
